# Unveiling the potential use of bioprinting materials in directing stem cell fate for cartilage regeneration: Focusing on induced pluripotent stem cells and enhanced lubrication (Review)

**DOI:** 10.3892/mi.2026.317

**Published:** 2026-04-24

**Authors:** Gunasekara Wijesinghege Nimanthi Kaushalya, Sanath Rajapakse

**Affiliations:** Department of Molecular Biology and Biotechnology, Faculty of Science, University of Peradeniya, Peradeniya 20400, Sri Lanka

**Keywords:** 3D bioprinting, bioink, lubrication, induced pluripotent stem cells, chondrocyte differentiation

## Abstract

Cartilage is a strong, yet flexible, type of connective tissue found in various parts of the body. Cartilage that covers the ends of bones in joints, referred to as articular cartilage, is frequently damaged during injuries and osteoarthritis (OA). Lack of blood vessels and nerves in the cartilage limits its self-renewal capacity, rendering cartilage regeneration a critical challenge. Three-dimensional (3D) bioprinting provides a promising approach for cartilage regeneration by depositing bioink layer by layer to create a 3D structure of more complex tissues such as cartilage, with clinically relevant sizes and mimicking the native shape and natural microenvironment. The present review discusses the potential of bioprinting materials to direct the fate of induced pluripotent stem cells (iPSCs) towards chondrocytes, while simultaneously enhancing lubrication. This will be achieved by incorporating iPSCs, natural polymers, synthetic polymers and lubricants such as lubricin, growth factors and signaling molecules that are involved in chondrogenesis. By optimizing bioink formulation and other parameters in bioprinters, the production of tissues in a precisely controlled manner is ensured, and limitations in current treatments for degenerative diseases such as OA can be avoided. Researchers have developed various complex and functional tissues and organs using bioprinting with successful animal trials, and are in the process of developing patient-specific iPSCs to generate personalized cartilage grafts having high and prolonged regenerative ability with further enhanced lubrication. The present review aimed to provide hope for patients suffering from cartilage degeneration by demonstrating the potential of 3D bioprinting for effective cartilage regeneration.

## 1. Cartilage: A closer look, cartilage degradation and the challenge of regeneration

Cartilage is a resilient, yet flexible connective tissue that performs several essential physiological functions, including the maintenance of structural integrity and preservation of shape, the absorption of mechanical shock during movement, and enabling smooth joint motion (1). There are three types of cartilage: Elastic cartilage, fibrocartilage and hyaline cartilage. Hyaline cartilage is the most abundant, which provides smooth, low-friction surfaces that facilitate joint movement. It is located at the ends of bones in synovial joints, and in structures such as the nasal septum. A specialized form of hyaline cartilage, known as articular cartilage, covers the articulating surfaces of bones within synovial joints and functions in close interaction with synovial fluid, which provides lubrication and facilitates smooth joint movement (2,3). Articular cartilage is composed of a complex extracellular matrix (ECM) that is rich in type II collagen fibrils embedded within a highly hydrated proteoglycan network, predominantly consisting of aggrecan (4).

**Figure 1:**
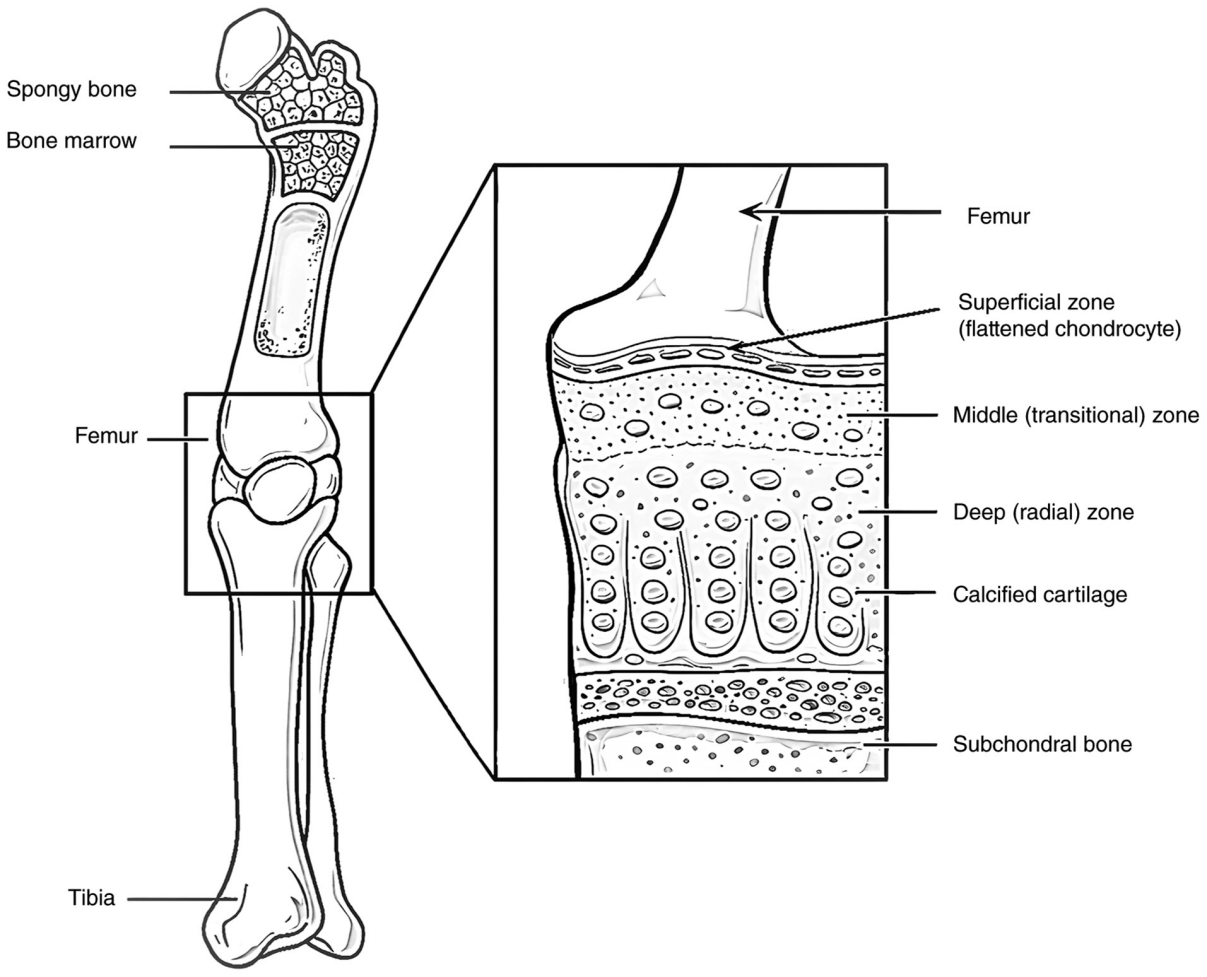
Anatomy of the leg: This image illustrates the long bones of the leg, highlighting the smooth articular cartilage covering the joint surfaces and the presence of red bone marrow within the spongy bone. The information presented in the image was obtained from previous studies (6-10).

This unique structure confers exceptional biomechanical properties to articular cartilage, including low friction, high compressive strength and marked resilience (5). Its smooth, lubricated surface enables near-frictionless joint articulation, while its ability to withstand substantial mechanical loads protects the underlying subchondral bone from mechanical damage (4). Articular cartilage contains chondrocytes, the only resident cell type, which are embedded within an ECM that they synthesize, remodel and maintain. These cells are distributed throughout the cartilage matrix in a distinct spatial organization, as illustrated in Fig. 1 (6-10). Notably, articular cartilage is characterized as an avascular and aneural tissue that significantly limits its intrinsic capacity for repair following injury (3,11,12). Consequently, damage to articular cartilage rarely heals spontaneously and often leads to the progressive deterioration of joint structure and function over time (6).

The limited regenerative capacity of articular cartilage renders it particularly susceptible to injury and degenerative joint diseases, such as osteoarthritis (OA). Repetitive mechanical loading and age-related wear can initiate cartilage degradation, leading to progressive structural and functional deterioration. As degeneration advances, the protective cartilage layer becomes progressively thinner, eventually exposing the underlying subchondral bone to direct mechanical stress. This process accelerates joint degeneration and ultimately contributes to the development and progression of OA (12). OA, the most common form of arthritis, is characterized by the progressive degeneration of articular cartilage and alterations in subchondral bone, leading to chronic pain, joint stiffness and reduced mobility.

Despite extensive research efforts, currently available medical interventions have not reliably achieved the complete regeneration of damaged cartilage nor provided consistent long-term restoration of joint function. Consequently, considerable attention has been directed towards the development of regenerative therapeutic strategies. Emerging approaches, including stem cell transplantation combined with biocompatible scaffolds and bioactive growth factors, have been widely investigated in preclinical and clinical settings, with promising results reported from *in vitro* studies, animal models and early clinical applications (13-18). Conventional surgical techniques such as osteochondral plug grafting and subchondral bone microfracture, which stimulate the recruitment of endogenous bone marrow (BM)-derived cells have demonstrated some effectiveness in treating small focal cartilage defects. However, these approaches often fail to provide durable repair in cases of extensive cartilage loss associated with degenerative conditions such as OA (17,18). Stem cell-based therapies provide significant promise by introducing regenerative cells capable of promoting cartilage repair, reducing inflammation, and alleviating pain, particularly where conventional therapies are insufficient.

## 2. Stem cells and induced pluripotent stem cells

Stem cells are characterized by their capacity for self-renewal and multilineage differentiation (10). Based on their differentiation potential, stem cells are classified as unipotent, multipotent and pluripotent (19). Pluripotent stem cells (PSCs), including embryonic stem cells (ESCs) and induced PSCs (iPSCs) can differentiate into all somatic cell types (20). ESCs are derived from the inner cell mass of blastocysts, whereas iPSCs are generated through the reprogramming of somatic cells, such as fibroblasts, via the enforced expression of defined transcription factors. This landmark discovery, recognized by the 2012 Nobel Prize in Physiology or Medicine (https://www.nobelprize.org/prizes/medicine/2012/summary/), revolutionized regenerative medicine. Cellular reprogramming typically involves the transcription factors OCT4, SRY-related HMG-box (SOX)2, KLF4 and c-Myc, which restore pluripotency by resetting gene expression patterns. Although reprogramming efficiency remains relatively low, successfully generated iPSCs exhibit morphological, molecular and functional characteristics comparable to those of ESCs. Their pluripotent status is commonly confirmed through teratoma formation assays demonstrating differentiation into tissues derived from all three germ layers (21).

Human PSCs (hPSCs) represent a promising source of chondroprogenitor cells owing to their unlimited proliferative capacity and broad differentiation potential (22,23). In addition to PSCs, mesenchymal stem cells (MSCs) have been extensively investigated for cartilage repair. MSCs can be isolated from multiple tissues, including BM, adipose tissue, synovium and cartilage. They exhibit inherent chondrogenic potential with the capacity for *in vitro* expansion (24). Of note, articular cartilage progenitor cells (ACPCs) have been identified as a resident progenitor cell population with potential relevance for cartilage regeneration (25-27). ACPCs predominantly localize in the superficial zone of articular cartilage and exhibit key stem cell-like characteristics, including clonogenicity, migratory capacity and chondrogenic differentiation potential (25,27). Compared with fully differentiated chondrocytes, ACPCs demonstrate enhanced proliferative capacity, while maintaining a stable articular cartilage phenotype, rendering them a promising cell source for regenerative strategies (26,27). Recent research has highlighted their potential role in maintaining cartilage homeostasis and contributing to tissue repair, particularly during the early stages of OA (28). However, despite their therapeutic promise, MSC-based cartilage repair approaches are frequently complicated by phenotypic instability, including a tendency toward hypertrophic differentiation and endochondral ossification (29).

## 3. Chondrogenesis

**Figure 2:**
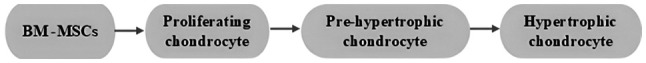
Chondrogenesis: The phases of chondrogenesis are illustrated, beginning with MSCs and ending with the development of hypertrophic chondrocytes. The information presented in the image was obtained from previous studies (4,31). MSCs, mesenchymal stem cells; BM, bone marrow.

Chondrogenesis is a tightly regulated developmental process through which MSCs differentiate into chondrocytes, the specialized cells responsible for producing and maintaining the ECM of cartilage. This process involves sequential events, including mesenchymal condensation, proliferation and the activation of cartilage-specific gene expression programs (Fig. 2) (30). The transcription factor, SOX9, is widely recognized as the master regulator of chondrogenesis (31). During differentiation, MSCs undergo characteristic morphological changes and initiate the expression of key cartilage ECM components, including type II collagen and aggrecan (4). Although these molecules are not exclusively restricted to cartilage, they represent the principal structural constituents responsible for the unique mechanical and functional properties of cartilage tissue. Chondrogenesis plays a fundamental role in embryonic skeletal development and continues to be essential for the maintenance, homeostasis, and repair of cartilage throughout life.

## 4. Proliferation and pre-hypertrophic differentiation

BM-MSCs undergo staged differentiation toward mature chondrocytes. The initial proliferative phase ensures sufficient cell expansion (32). Subsequently, cells enter a pre-hypertrophic stage marked by the upregulation in the levels of chondrogenic markers, including aggrecan, Col2a1 and SOX9, alongside the suppression of mesenchymal markers (33). ECM synthesis is initiated during this phase.

## 5. Hypertrophic differentiation and endochondral ossification

The differentiation of BM-MSCs into hypertrophic chondrocytes represents the final stage of chondrogenic maturation. During this phase, the cells undergo further phenotypic maturation accompanied by distinct alterations in gene expression. Hypertrophic chondrocytes are characterized by the markedly elevated expression of type X collagen, alkaline phosphatase and vascular endothelial growth factor (VEGF) (34). Type X collagen is widely recognized as a hallmark marker of hypertrophic chondrocytes and contributes to the structural organization of the developing cartilage matrix. Increased alkaline phosphatase activity, an enzyme closely associated with matrix mineralization, indicates the onset of the transition toward ossification. In addition, VEGF promotes vascular invasion, a critical event in the later stages of endochondral ossification. This tightly regulated process involves the gradual replacement of cartilage with bone and plays an essential role in normal skeletal development. Under physiological conditions, hypertrophic differentiation is carefully controlled to preserve the stability and functional integrity of the cartilage matrix (35). Consequently, a comprehensive understanding of the molecular and cellular mechanisms governing this differentiation process is crucial for the development of effective therapeutic strategies for cartilage regeneration and repair.

Early studies have demonstrated that hPSCs can generate MSC-like cells through various approaches, including co-culture with OP9 stromal cells (36), spontaneous differentiation under standard culture conditions (37), and direct mesengenesis under modified culture conditions (38,39). These strategies yielded mesenchymal-like cells that often required subsequent expansion in conventional MSC culture media to acquire tri-lineage differentiation potential characteristic of MSCs (40,41). Alternative methods, such as treatment with the small molecule inhibitor, SB431542, have also been used to produce a mesenchymal cell population. However, these cells frequently exhibited heterogeneous developmental origins and similarly required expansion in MSC-supportive media to attain MSC-like properties (42). Although these approaches have shown promise, the *in vitro* chondrogenic capacity of PSC-derived MSCs has generally been inferior to that of adult MSCs, particularly when transforming growth factor-β (TGF-β) alone was used to induce chondrogenesis (43). Comparative analyses of human BM-MSCs and iPSC-derived MSCs from the same donor have revealed significant functional differences, with iPSC-derived MSCs demonstrating reduced chondrogenic potential (44,45).

## 6. MSC therapy for cartilage regeneration

Recent advances in regenerative medicine have increasingly focused on cell- and tissue-engineering strategies that utilize articular chondrocytes or chondrogenic adult stem cells (ASCs), such as MSCs derived from BM or adipose tissue (46,47). In established clinical approaches, selected cells are implanted into the defect site using a periosteal patch in autologous chondrocyte implantation (ACI) or a collagen membrane in matrix-induced ACI, to improve therapeutic outcomes (18). Cell-based therapies, specific stem cell populations such as BM-MSCs, which possess the capacity to differentiate into chondrocytes, the principal cell type responsible for cartilage matrix production, are first isolated and selected. These cells are subsequently expanded *in vitro* under growth factor-supplemented conditions to generate sufficient numbers for therapeutic use. *Ex vivo* expansion is often required to achieve an adequate yield for effective treatment. However, prolonged culture and expansion can alter cellular phenotype and functional properties, thereby diminishing their suitability for cartilage repair (48). Notably, *in vivo* studies have demonstrated that uncultured or minimally expanded articular chondrocytes are more capable of generating stable, articular-like permanent cartilage compared with extensively cultured cells (49,50). This observation underscores the importance of preserving the native phenotype and functional characteristics of therapeutic cells during preparation for clinical use.

## 7. Use of iPSCs for cartilage regeneration

The use of ESCs and ASCs, although promising for cartilage regeneration, is associated with several critical limitations. ESCs are derived from the inner cell mass of embryos, and their isolation requires the destruction of the embryo, raising substantial ethical concerns (51). Moreover, ESC-based therapies carry a risk of teratoma formation as residual undifferentiated cells may give rise to tumors following transplantation (52). ASCs, while more readily accessible from sources such as BM and adipose tissue, possess limited proliferative capacity and may undergo phenotypic drift during *ex vivo* expansion, potentially compromising their chondrogenic potential (47). Furthermore, achieving the consistent and reproducible differentiation of both ESCs and ASCs into functional chondrocytes remains challenging, as it requires precise control of culture conditions and often involves complex combinations of growth factors and biomaterials.

iPSCs provide several distinct advantages for cartilage regeneration. Unlike ESCs, iPSCs can be generated from the somatic cells of patients themselves, such as dermal fibroblasts, eliminating ethical concerns related to embryo destruction (1). This patient-specific approach also reduces the risk of immune rejection, enhancing the feasibility of clinical translation (53). Additionally, iPSCs exhibit broad differentiation potential and can be directed toward chondrogenic lineages, including the generation of functional chondrocytes (54). Their plasticity enables the development of personalized cartilage grafts for transplantation, representing a promising strategy for treating cartilage defects and degenerative diseases, such as OA.

Despite these advantages however, several challenges need to be addressed before the widespread clinical application of iPSC-based therapies. A crucial unresolved question is whether disease-associated abnormalities present in patients with inflammatory joint disorders, such as OA or rheumatoid arthritis, may be retained in iPSCs and their chondrocyte derivatives (55). Addressing this issue will require comprehensive comparative analyses of patient-derived cells, together with the development of optimized culture and differentiation protocols to ensure therapeutic safety and efficacy.

**Figure 3:**
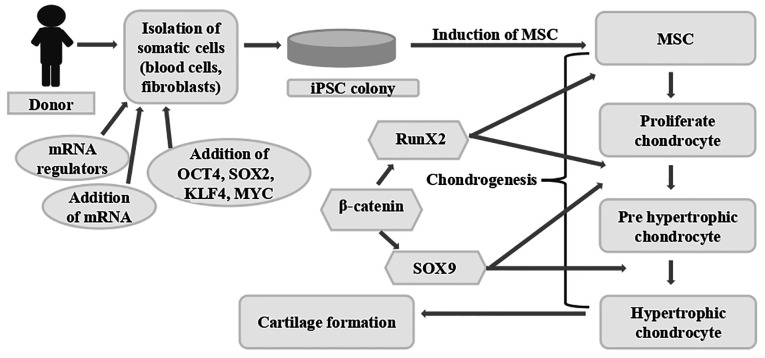
Diagrammatic illustration of cartilage regeneration using iPSCs. By introducing particular transcription factors, somatic cells can be transformed into iPSCs. These iPSCs have the potential to develop into cartilage-producing chondrocytes. The procedure for creating cartilage tissue from cells iPSCs is shown in this schematic. Certain transcription factors, including OCT4, SOX2, KLF4 and Myc, are introduced into the somatic cells of an individual (fibroblasts, for example) to reprogram them into iPSCs. These iPSCs can differentiate into a variety of cell types, including chondrocytes, which are the cells that form cartilage. A series of signaling events and the activation of important transcription factors coordinate the differentiation process. An early transcription factor called Runx2 is essential for starting chondrogenesis. Subsequently, genes involved in the synthesis of cartilage matrix, including collagen type II and aggrecan, are expressed in response to SOX9, another essential transcription factor. Following a maturation process, the differentiated chondrocytes go from proliferative to pre-hypertrophic and ultimately hypertrophic. The information presented in the image was obtained from previous studies (31,32,35,57-65). iPSCs, induced pluripotent stem cells.

**Figure 4:**
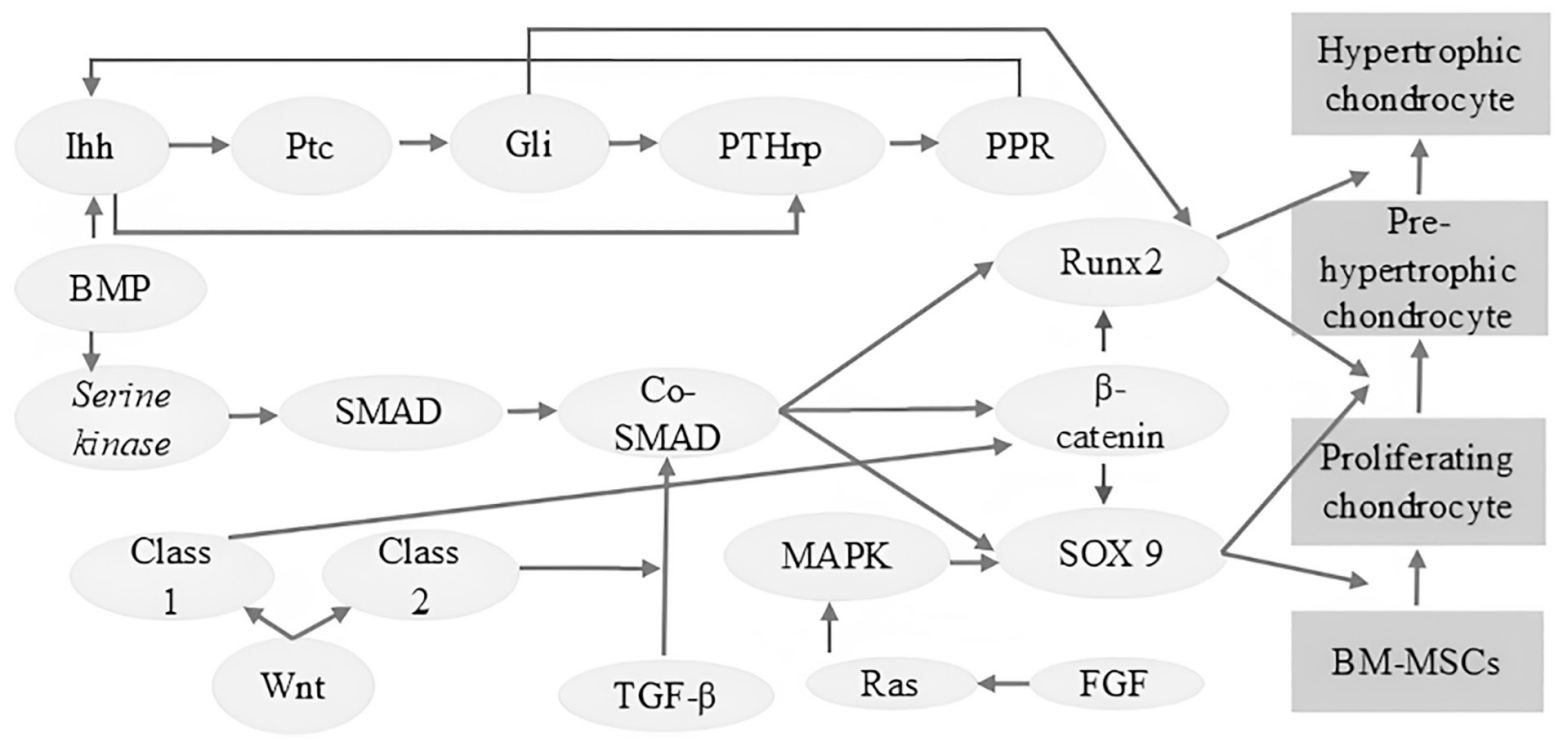
Schematic illustration of the complex interplay of signaling pathways for chondrocyte differentiation. The complex network of signaling pathways that control the differentiation of BM-MSCs into chondrocytes is simplified in the image. There are several phases in this developmental process, such as pre-hypertrophy, hypertrophy and proliferation. These phases are carefully coordinated by a wide range of signaling pathways, including the Wnt/β-catenin pathway, which stimulates chondrocyte proliferation and differentiation; the TGF-β/SMAD pathway, which is essential for chondrogenesis as it controls the production of ECM and chondrocyte maturation; the BMP/SMAD pathway, which stimulates chondrocyte differentiation and controls matrix synthesis; the Ihh signaling pathway and PTHrP signaling pathway, which controls chondrocyte proliferation, hypertrophy and endochondral bone formation; the FGF signaling pathway, which stimulates both chondrocyte proliferation and differentiation; and the MAPK signaling pathway, which is involved in multiple chondrogenesis, including proliferation, differentiation and matrix synthesis. It is important to understand that these signaling pathways show complex interactions, with feedback loops and crosstalk having a significant impact on chondrocyte development. Developing successful strategies for cartilage tissue engineering and repair requires a thorough understanding of these signaling mechanisms (35,61,64,65). BM-MSCs, bone marrow-derived mesenchymal stem cells; TGF-β, transforming growth factor-β; Ihh, Indian hedgehog; PTHrP, parathyroid hormone-related peptide; FGF, fibroblast growth factor.

For the successful clinical application of PSCs in joint cartilage regeneration, several key objectives need to be achieved. These include the efficient generation of lineage-specific chondroprogenitors from hPSCs, the expansion of these progenitors to clinically relevant numbers, without compromising their chondrogenic capacity and their differentiation, either *in vitro* or *in vivo*, into stable articular or meniscal (permanent) chondrocytes rather than transient growth plate chondrocytes (56). As illustrated in Fig. 3, iPSCs are derived from somatic cells such as fibroblasts and subsequently differentiated into MSC-like intermediates prior to cartilage formation. Chondrocyte differentiation is governed by complex and tightly interconnected signaling pathways, as depicted in Figs. 3 and 4 (31,32,35,57-65).

The advantages and limitations of commonly used cell sources for cartilage bioprinting are summarized in Table I.

## 8. Signaling pathways for chondrocyte differentiation

SOX9 signaling, Wnt signaling, bone morphogenetic protein (BMP) signaling, Indian Hedgehog (Ihh) signaling, parathyroid hormone-related peptide (PTHrP) signaling, TGF-β signaling, fibroblast growth factor (FGF) signaling and Runx Family transcription factors are involved in chondrocyte differentiation (34,57,60,61). In addition, adenosine signaling, oxygen tension and reactive oxygen species are involved in chondrocyte development (60).

SOX9, a member of the SRY-related HMG-box (SOX) family of transcription factors, is a master regulator of chondrogenesis, the process of cartilage formation. It plays a pivotal role in orchestrating the differentiation of MSCs into chondrocytes, the specialized cells that produce the ECM of cartilage. SOX9 functions as an early marker of chondrogenic commitment, initiating a cascade of events that leads to the expression of genes essential for cartilage development, such as collagen type II, collagen type IX and aggrecan. Furthermore, SOX9 plays a dual role in chondrocyte maturation, promoting early chondrocyte hypertrophy while simultaneously inhibiting excessive hypertrophy, a crucial step in endochondral bone formation. This intricate balance is achieved through complex interactions with other transcription factors, including SOX5 and SOX6, and by modulating various signaling pathways, such as Wnt, TGF-β and BMP. Numerous skeletal disorders have been linked to the dysregulation of SOX9 expression or activity, underscoring the vital role of the protein in preserving skeletal health and cartilage homeostasis (30,31,57,60).

Wnt signaling influences skeletal development through multiple mechanisms. β-catenin serves as the central mediator of canonical Wnt signaling, which inhibits chondrocyte differentiation while promoting osteoblast differentiation. By contrast, non-canonical Wnt signaling pathways, involving factors such as Wnt5a and Wnt11, regulate chondrocyte proliferation and columnar organization and can also promote chondrocyte differentiation. Wnt signaling further contributes to the spatial orientation of chondrocytes within the growth plate, thereby supporting proper skeletal patterning. The coordinated interplay between canonical and non-canonical Wnt pathways maintains the balance between chondrogenesis and osteogenesis, ensuring normal skeletal development and homeostasis (30,60).

Ihh is a key regulatory molecule in endochondral ossification. It controls chondrocyte proliferation and maturation through both PTHrP-dependent and PTHrP-independent mechanisms. Ihh stimulates PTHrP expression, which in turn delays chondrocyte hypertrophy. Independent of PTHrP, Ihh directly promotes chondrocyte proliferation through the activation of GLI family transcription factors. The overexpression of PTHrP results in delayed chondrocyte differentiation, whereas the deletion of PTHrP leads to premature chondrocyte maturation at inappropriate anatomical locations. Through these coordinated mechanisms, Ihh ensures proper chondrocyte proliferation, differentiation and subsequent bone formation during endochondral ossification (60).

FGFs are essential signaling molecules in skeletal development (61,62). FGF receptors (FGFR1, FGFR2 and FGFR3) are expressed at various stages of chondrocyte development and regulate both proliferation and hypertrophy (62-64). Among the FGF ligands, FGF9 and FGF18 play particularly critical roles in chondrogenesis. FGF9 promotes early chondrocyte proliferation and hypertrophy, and also contributes to angiogenesis (62,64). In later developmental stages, FGF18 exhibits a dual function, initially stimulating proliferation and hypertrophy, followed by suppression of proliferation and delayed hypertrophy (60).

BMPs are critical regulators of chondrogenesis. BMP signaling is mediated through the receptors BMPR1 and BMPR2, the latter possessing serine/threonine kinase activity. The activation of BMP receptors induces the phosphorylation of SMAD transcription factors, which then form complexes with co-SMADs and translocate to the nucleus to regulate the expression of target genes, including Runx2, SOX9 and β-catenin. β-catenin can, in turn, modulate the expression of SOX9 and Runx2, both of which are essential for chondrogenesis. In addition to SOX9, SOX5 and SOX6 are also required for proper chondrocyte differentiation. SOX9 is particularly critical during early chondrogenesis, as it promotes the expression of genes involved in cartilage matrix synthesis. The importance of BMP signaling in endochondral ossification is highlighted by evidence indicating that the inhibition of BMP signaling or genetic deletion of BMP receptors results in impaired chondrocyte formation and maturation (60).

Runx2 and Runx3, members of the Runx transcription factor family, are essential regulators of chondrocyte hypertrophy. Runx2 plays a central role by directly inducing Ihh expression, interacting with BMP-regulated SMADs, and activating hypertrophic marker genes. Runx3 deficiency results in a relatively mild phenotype, whereas Runx2 deficiency markedly impairs chondrocyte hypertrophy. However, the simultaneous absence of both Runx2 and Runx3 completely abolishes chondrocyte maturation, demonstrating their complementary and indispensable roles in this developmental process. Runx2 is particularly involved in the later stages of chondrogenesis and contributes to hypertrophic differentiation (34,60).

In contrast to adult BM-MSCs, which require TGF-β for maintenance in defined culture conditions (65), PSC-derived chondrogenic cells, such as those derived from neural crest-like progenitors, may exhibit distinct growth regulatory mechanisms. When expanded in the presence of SB431542, these cells demonstrated sustained chondrogenic activity and reduced dependence on TGF-β signaling (66). This finding suggests that TGF-β may function as an intrinsic regulatory ‘brake’ on the proliferation of certain PSC-derived embryonic cells, similar to its established role in controlling endothelial cell growth (67) and hematopoietic progenitor proliferation (68). Notably, although both PSC-derived ectomesenchymal cells and BM-MSCs have been proposed to share neural crest origins (69-71), their differential responses to TGF-β signaling indicate fundamental differences in their growth characteristics and differentiation potential.

## 9. Influence of 2D and 3D cell culture systems on chondrogenesis

Cell culture conditions play a critical role in regulating cellular phenotype, differentiation potential and ECM production during cartilage tissue engineering. Traditional two-dimensional (2D) monolayer culture systems are widely used for cell expansion due to their simplicity and ease of manipulation. However, these systems often fail to preserve the native chondrocytic phenotype. Chondrocytes expanded in 2D culture frequently undergo dedifferentiation, which is characterized by the acquisition of a fibroblast-like morphology and reduced expression of cartilage-specific markers such as type II collagen and aggrecan, accompanied by increased expression of fibrocartilaginous markers, including type I collagen (72,73). Similarly, prolonged expansion of MSCs under 2D culture conditions may alter their gene expression profiles and diminish their subsequent chondrogenic differentiation potential.

In contrast, two-dimensional (3D) culture systems including pellet cultures, hydrogels and scaffold-based matrices more closely mimic the native cartilage microenvironment by facilitating spatial cell-cell and cell-matrix interactions. These interactions are essential for mesenchymal condensation and the activation of chondrogenic transcriptional programs regulated by factors, such as SOX9. Cells cultured in 3D environments typically adopt a rounded morphology similar to native chondrocytes and exhibit enhanced expression of cartilage-specific genes, including COL2A1, aggrecan and SOX9, as well as increased deposition of ECM components, such as glycosaminoglycan (GAG) and type II collagen (74,75). Moreover, 3D culture conditions promote more physiologically relevant biochemical and biomechanical cues, enabling the development of tissue constructs with structural and functional properties closer to native cartilage. For these reasons, 3D culture platforms are widely regarded as more suitable for cartilage regeneration studies. They are often integrated with emerging technologies such as 3D bioprinting to generate biomimetic cartilage constructs.

## 10. 3D bioprinting

Bioprinting is an emerging technology that utilizes bioinks, composed of biomaterials and living cells to fabricate 3D tissue constructs for regenerative medicine. These bioinks should possess appropriate mechanical and rheological properties to ensure printing accuracy, structural stability and the functional performance of the resulting tissues (71). Biofabrication is a broader and rapidly evolving field that focuses on the generation of biologically functional constructs with hierarchical organization (76,77). It encompasses a wide range of technologies used to create biological substitutes, including conventional tissue engineering strategies, such as scaffold fabrication and cell seeding. Within this framework, bioprinting represents a specialized subset of biofabrication that specifically refers to the layer-by-layer fabrication of 3D tissue and organ constructs using cell-laden bioinks.

3D bioprinting, a key modality within biofabrication, enables the production of constructs with precise spatial resolution, defined geometry and tunable mechanical properties. This technique allows for the accurate placement of biomaterials, cells and biological cues in a controlled, layer-by-layer manner, rendering it a promising strategy for the development of personalized regenerative implants (78). Articular cartilage, a thin and avascular connective tissue, represents a relatively suitable target for bioprinted regenerative therapies compared with highly vascularized organs. Due to its low cell density and the lack of vascular supply, cartilage has limited intrinsic regenerative capacity (79). Consequently, untreated articular cartilage injuries often progress to OA (80).

Bioprinting technologies rely on computer-aided design models to define the architecture of printed constructs. Medical imaging modalities, including computed tomography (CT), magnetic resonance imaging (MRI) and X-ray imaging, provide detailed anatomical information that can be converted into high-resolution 2D image slices and subsequently reconstructed into 3D digital models suitable for additive manufacturing and stereolithography-based applications. Such 3D-printed anatomical models are widely used in surgical planning and medical education, particularly for complex anatomical structures. Overall, the major advantages of bioprinting include high reproducibility, precise control over construct architecture, and the potential for scalable and high-throughput production. These features position bioprinting as a promising platform in tissue engineering and regenerative medicine for generating functional tissue substitutes. Bioprinting technologies are generally classified into four principal categories: Extrusion-based bioprinting, inkjet bioprinting, laser-assisted bioprinting and stereolithography-based bioprinting (76,81-85). Each technique operates through distinct mechanisms and provides specific advantages and limitations in terms of printing resolution, cell viability and material compatibility.

Extrusion-based bioprinting is the most widely applied technique in cartilage tissue engineering due to its ability to process high-viscosity bioinks and cell-laden hydrogels. In this approach, bioinks are extruded through a nozzle using pneumatic, piston, or screw-driven pressure to generate continuous filaments that are deposited layer by layer to form 3D structures. This method supports a wide range of biomaterials and enables printing at relatively high cell densities. However, shear stress generated during extrusion may negatively affect cell viability, and the printing resolution is typically limited by the nozzle diameter (86).

Inkjet bioprinting deposits bioink droplets through thermal or piezoelectric actuation in a non-contact manner. This approach provides a high printing speed and good spatial resolution; however, it is generally restricted to low-viscosity bioinks and relatively low cell densities, which may limit its ability to generate mechanically robust cartilage constructs. Laser-assisted bioprinting utilizes laser pulses to create localized pressure that propels droplets of bioink onto a receiving substrate without the use of a nozzle. This nozzle-free technique minimizes mechanical stress on cells and enables high spatial precision with excellent cell viability. However, the high cost and technical complexity of the system limit its widespread application (87).

Stereolithography-based bioprinting relies on light-induced photopolymerization to solidify photosensitive bioinks layer by layer, enabling the fabrication of constructs with high structural fidelity and resolution. Nevertheless, this method is typically limited to photo-crosslinkable biomaterials and may not be suitable for bioinks containing very high cell densities. Among these techniques, extrusion-based bioprinting remains the most commonly used approach in cartilage tissue engineering because it allows the deposition of high-viscosity hydrogel bioinks containing chondrocytes or mesenchymal stem cells, which are essential for generating mechanically stable and biologically functional cartilage constructs.

## 11. Bioink

3D bioprinting utilizes bioinks, which are printable biomaterials designed for the fabrication of living tissues. Although numerous biomaterials have been investigated for the repair of diseased or damaged tissues, the majority remain incompatible with current bioprinting technologies (88). Bioinks are broadly categorized into two main types: Scaffold-based and scaffold-free systems. Scaffold-free bioinks mimic aspects of embryonic development and neo-tissue formation. This strategy relies on tissue spheroids, cell pellets, or tissue strands to generate large-scale functional constructs without the use of an exogenous scaffold (89). By contrast, scaffold-based bioinks incorporate cells within supportive matrices such as hydrogels, microcarriers, or decellularized ECM components. These matrices provide structural support and promote cell proliferation, differentiation and tissue maturation. Various bioprinting techniques have been developed to optimize print fidelity and spatial resolution when using these bioink systems (76).

Initially, additive manufacturing technologies were developed for non-biological applications. Common materials used in traditional 3D printing, including metals, ceramics and thermoplastic polymers, are generally unsuitable for biological applications as they often require high temperatures, organic solvents, or harsh crosslinking conditions that are incompatible with living cells. One of the greatest challenges in fabricating functional tissues and organs using 3D printing lies in replicating the mechanical, chemical and morphological properties of native tissues. Bioinks play a critical role in overcoming these challenges by protecting encapsulated cells from mechanical stress during extrusion and from adverse environmental conditions during the printing process (90).

An ideal bioink should possess several essential characteristics, including printability, high mechanical integrity, structural stability, resistance to dissolution in culture media, a biodegradation rate compatible with tissue regeneration, non-toxicity, non-immunogenicity, and the capacity to promote cell adhesion and proliferation. Additionally, bioink materials should be cost-effective and amenable to scalable manufacturing (91). Over the past decade, 3D printing technologies have rapidly expanded and become a cornerstone of tissue engineering research (92). A wide range of materials including ceramics, polymers, elastomers, hydrogels and lipids have been explored as bioinks for fabricating 3D constructs. Advanced bioinks can be classified into five main categories: Multi-material bioinks, stimuli-responsive bioinks, self-assembling bioinks, biomolecular bioinks and nanoengineered bioinks, many of which exhibit shear-thinning properties that facilitate extrusion-based printing (76).

In recent years, scaffold-based and scaffold-free bioinks have emerged as the two principal strategies in bioprinting. Scaffold-based systems consist of biomaterials combined with cells that are co-deposited to form a construct, with the biomaterial serving as structural support to promote cell proliferation and differentiation (6,93). By contrast, scaffold-free systems rely on cellular aggregates, such as spheroids, tissue strands or cell pellets that secrete ECM-like components to maintain structural integrity (94). Although scaffold-based approaches remain the most widely used, both strategies present distinct advantages and limitations.

The functional performance of bioprinted tissues depends on the physicochemical and biological properties of the bioink. For cartilage tissue engineering in particular, bioinks should meet both material and biological requirements. Key characteristics include appropriate printability, bioresorbability and biodegradability (6). Additional desirable features include high printing resolution, cost-effectiveness, industrial scalability and rapid post-printing maturation. In hydrogel-based systems, other key attributes include reversible gelation (facilitating pre-culture prior to implantation), rapid gelation kinetics and minimal volumetric changes during crosslinking (95).

Successful 3D cell printing also requires bioinks with optimal viscosity to ensure smooth extrusion, while maintaining structural fidelity after deposition. Following printing, the material must undergo rapid stabilization, typically through solvent evaporation or polymer crosslinking to maintain the intended 3D architecture. Notably, bioinks must remain cytocompatible and non-toxic throughout both the printing and crosslinking processes. Although certain robust materials require high temperatures or toxic solvents for processing, such conditions are generally incompatible with cell-laden bioprinting applications (96).

In stem cell bioprinting, the preservation of cell viability during printing and provision of a supportive microenvironment for post-printing growth and differentiation are essential. This requirement typically favors aqueous, hydrogel-based bioinks. Both natural and synthetic polymers are widely used. Natural ECM-derived polymers such as collagen, fibrin and gelatin are particularly attractive due to their inherent biocompatibility and ability to mimic the native cellular microenvironment. Other natural polymers used as bioinks include chitosan and alginate. Synthetic polymers, such as Pluronic F127, polyethylene oxide and polyethylene glycol (PEG), provide tunable mechanical and physicochemical properties. While natural polymers provide excellent biocompatibility and cell-supportive properties, they often exhibit limited mechanical strength and structural stability. Therefore, they are frequently combined with synthetic polymers to enhance versatility, stability and mechanical performance in 3D bioprinting applications (86).

Bioinks designed for stem cell-based 3D bioprinting need to achieve a balance between printability, biocompatibility, and the capacity to support cell growth and function. Although natural polymers, such as alginate, chitosan, agarose, hyaluronic acid (HA) and fibrin provide strong biocompatibility, they may require chemical or physical modification to improve mechanical strength or printing resolution. For instance, alginate undergoes rapid gelation in the presence of calcium ions, rendering it suitable for high-throughput bioprinting (94,97-100). Fibrin provides rapid crosslinking but has relatively low mechanical strength and is often blended with other polymers to enhance structural stability. Synthetic polymers, such as Pluronic F127 and PEG provide adjustable properties and can be chemically modified for specific applications. Ultimately, the choice and composition of bioink materials should be tailored to the specific cell type and target tissue being engineered (96,100).

Natural and synthetic hydrogels are widely used as bioink materials in cartilage tissue engineering due to their ability to mimic the hydrated ECM environment required for chondrocyte survival and differentiation. Natural biomaterials, such as alginate, gelatin, collagen, HA, agarose and chitosan are particularly attractive as they exhibit excellent biocompatibility, bioactivity and structural similarity to native cartilage ECM. These materials often contain inherent cell-binding motifs that support cell adhesion, proliferation and chondrogenic differentiation. For example, alginate is widely used due to its rapid ionic crosslinking and ability to maintain the rounded morphology of chondrocytes, which is critical for preserving their phenotype. Similarly, gelatin- and collagen-based hydrogels provide cell-recognition sites that enhance cell attachment and ECM deposition during cartilage formation (76,91).

However, natural hydrogels also present several limitations, including relatively weak mechanical strength, rapid degradation and batch-to-batch variability. These drawbacks can compromise the structural integrity of printed constructs, particularly when engineering load-bearing tissues such as articular cartilage. In addition, although the majority of natural polymers are biocompatible, certain materials may trigger mild immune responses depending on their source and purification process (101,102).

Synthetic polymers such as PEG, polycaprolactone (PCL) and pluronic-based hydrogels have therefore been explored to overcome these limitations. Synthetic bioinks provide highly tunable mechanical properties, controlled degradation rates and improved printability, rendering them suitable for fabricating mechanically stable constructs. PEG-based hydrogels, for example, allow for the precise modulation of stiffness and crosslinking density, which can influence stem cell fate and chondrogenic differentiation. Nevertheless, synthetic biomaterials generally lack intrinsic bioactive signals required for cell adhesion and matrix production, often necessitating modification with bioactive peptides or blending with natural polymers to enhance cellular responses (90,102). Hybrid bioinks that combine natural and synthetic components such as gelatin methacrylate (GelMA), alginate-gelatin blends and HA-PEG systems are therefore increasingly investigated as promising candidates for cartilage regeneration as they provide a balance between mechanical stability, printability and biological functionality.

## 12. Lubrication

The mucinous glycoprotein lubricin, encoded by the PRG4 gene is a principal boundary lubricant in diarthrodial joints. Lubricin is an elongated molecule (~200 nm in length and a ~1-5 nm in width) localized to the superficial zone of articular cartilage and present in synovial fluid (103-105). Its role in cartilage lubrication was first proposed by Radin *et al* (106) in 1970, and the term ‘lubricin’ was coined in 1981 following its identification as a mucin-like glycoprotein (107). Data from the initial study by Radin *et al* (106) demonstrated that a lubricin solution alone (without HA) reduced the coefficient of friction by ~2-fold compared with saline. Subsequent studies consistently confirmed that lubricin is a key mediator of boundary lubrication in synovial joints (108-114). Lubricin deficiency has been associated with severe joint disorders, including camptodactyly-arthropathy-coxa vara-pericarditis syndrome, which is characterized by accelerated joint degeneration. In such cases, lubrication supplementation has been shown to attenuate disease progression (112,115).

Cartilage degeneration typically begins at the superficial zone and progresses to deeper layers, including the subchondral bone, representing an early event in the pathogenesis of OA (4,116). Articular cartilage regeneration, particularly through human iPSC-derived cartilage, represents a rational patient-specific strategy that may overcome several limitations of conventional therapies (117). The development of 3D bioprinting technologies has further accelerated progress in this field.

3D bioprinting involves the encapsulation of cells, such as human iPSCs, human chondrocytes, or human MSCs within hydrogel-based bioinks (e.g., gelatin and alginate), and in some cases, nanomaterial-enhanced formulations (101,118). *Ex vivo* research has demonstrated the promising potential of 3D bioprinting for generating articular cartilage constructs (119), and various hydrogel systems have been evaluated *in vivo* to improve mechanical integrity and functional performance (120).

Targeting lubricin secretion has emerged as a major strategy for restoring the functional lubrication properties of regenerated cartilage, as native joint lubrication is highly dependent on PRG4 expression and lubricin production. Recent 3D bioprinting approaches have therefore focused on optimizing bioink formulations and encapsulated cells to enhance lubricin secretion (121,122). Lubricin functions as a protective molecule within synovial fluid, and recombinant lubricin has been shown to attenuate the onset of OA in experimental models (109,123). An optimized bioink formulation consisting of 14% GelMA and 2% oxidized methacrylated alginate has been identified as effective in promoting lubricin secretion while maintaining structural stability (122). This strategy may enhance the long-term success of cartilage regeneration. Further investigations are warranted to evaluate additional factors influencing lubrication, including biomaterial stiffness and growth factor incorporation. Moreover, iPSC-derived microtissues have been generated using nanofibrillated cellulose and alginate-based bioinks for *in vivo* mouse models, with modifications designed to stimulate aggrecan production, a key GAG involved in cartilage lubrication (124).

In addition to biological strategies, biomimetic material design has provided alternative approaches to restoring lubrication. A series of studies have described the synthesis of a polymer that mimics the distinctive bottlebrush architecture of lubricin, which is critical for its boundary lubrication function. This biomimetic polymer, synthesized from 2-hydroxyethyl acrylate and 2-methacryloyloxyethyl phosphorylcholine, effectively replaced natural lubricin when adsorbed onto damaged cartilage surfaces. The system achieved an extremely low coefficient of friction, comparable to that of native lubricin (125-127). These findings highlight the potential of advanced material design to generate high-performance, non-biological components for integration into bioprinted constructs and for improving the lubrication of damaged cartilage. Furthermore, *in vitro* research has demonstrated that gelatin-based hydrogels incorporating zwitterionic phosphocholine groups can enhance surface organization and improve native lubrication properties (128).

## 13. Comparison of 3D bioprinting with current treatments for OA and stem cell therapy

Bioprinting, an advanced technology that integrates biology with 3D printing, has transformed multiple medical fields, including drug delivery, tissue engineering and regenerative medicine. Bioprinted biomaterials provide a novel therapeutic strategy for OA, in contrast to conventional treatments, such as analgesic administration and joint replacement surgery, which are often associated with limited long-term efficacy and potential complications (129).

Several studies have demonstrated that stem cell therapy can promote cartilage regeneration with partial success (130). However, stem cell-based approaches present several limitations, including limited control over cell differentiation, poor cell survival and integration, and variability in stem cell activity that complicates the prediction of therapeutic outcomes and ethical concerns associated with the use of ESCs. ASCs, due to their multipotent nature, possess restricted differentiation capacity, whereas ESCs exhibit pluripotency and can differentiate into a broad range of cell types. Nevertheless, ethical concerns surrounding the use of ESCs have limited their clinical application.

To address these challenges, innovative strategies, such as the integration of 3D bioprinting with iPSCs have been proposed. iPSCs are generated by reprogramming mature somatic cells, such as dermal fibroblasts, into a pluripotent state. This approach provides a virtually unlimited and patient-specific cell source for regenerative applications, while avoiding the ethical concerns associated with ESCs (35,87,131,132).

Importantly, previous studies focusing specifically on cartilage tissue engineering have demonstrated improved outcomes when using 3D bioprinted constructs compared with conventional cell-based therapies, particularly in terms of enhanced cell viability, spatial organization and extracellular matrix production (133-137). For example, bioprinted cartilage constructs containing MSCs and hydrogel bioinks have been reported to achieve cell viability rates typically exceeding 80-90% after printing, while maintaining high chondrogenic differentiation capacity. In several *in vitro* and animal studies, bioprinted cartilage constructs have shown significant increases in GAG and collagen type II production compared with traditional scaffold-based or cell injection approaches (135,136,138). Furthermore, preclinical studies using bioprinted MSC-laden hydrogels have demonstrated cartilage defect filling and tissue regeneration efficiencies of almost 70-80%, whereas conventional microfracture or cell injection therapies often result in fibrocartilage formation and regeneration rates <40-50% (91,134,135). These findings highlight the improved structural organization and functional matrix production achieved through spatially controlled cell deposition in bioprinted constructs.

Furthermore, this technology enables the fabrication of complex 3D structures that closely replicate the architecture and microenvironment of native tissues. The precise spatial deposition of cells and biomaterials during bioprinting facilitates the generation of constructs that mimic the native joint microenvironment (102). By reproducing the intricate structure of affected cartilage, 3D bioprinting has the potential to promote tissue regeneration and potentially slow disease progression.

One of the most critical advantages of bioprinting is its capacity for personalized therapy (139). Bioprinted biomaterials can be tailored to the specific anatomical and physiological characteristics of each patient. By incorporating patient-specific parameters, such as joint geometry, defect size and disease severity, bioprinting enables the fabrication of customized constructs designed to optimize clinical outcomes (140). In addition, high-resolution imaging modalities, such as CT and MRI can be integrated into the bioprinting workflow to generate constructs that accurately replicate the geometry of cartilage defects and improve anatomical integration with host tissue (141-143)

3D bioprinting is also highly automated and scalable, enabling the relatively rapid fabrication of tissue constructs. Unlike conventional stem cell therapy, which often involves the transplantation of pre-differentiated chondrocytes, bioprinting allows stem cells within the printed construct to undergo proliferation and chondrogenic differentiation *in situ* following implantation. Bioinks typically consist of combinations of natural and synthetic polymers that provide a supportive ECM enriched with nutrients and growth factors. This microenvironment enhances cell viability and may promote more sustained and long-term regeneration compared with traditional cell injection approaches. Additionally, bioprinting allows for the spatial incorporation of therapeutic agents within the bioink, enabling the design of controlled and localized drug release profiles. These release patterns can be tailored to target areas of inflammation or cartilage degeneration according to patient-specific needs. Bioinks can also be engineered to replicate the mechanical properties of native cartilage, thereby providing structural support while facilitating tissue regeneration (144).

Despite its significant promise, 3D bioprinting in regenerative medicine continues to face important challenges. These include the optimization of bioink formulations, achieving adequate vascularization in larger constructs, addressing the technical complexity of bioprinting processes, and overcoming high production costs. Continued research and technological refinement are required to translate this promising approach into widespread clinical application.

## 14. Future directions

Future directions in cartilage bioprinting focus on developing techniques capable of generating more functional, durable and physiologically relevant tissue constructs. One critical strategy involves the use of multi-material bioprinting approaches to replicate the natural gradients present within cartilage. Native cartilage exhibits region-specific variations in mechanical properties, including stiffness and ECM composition. By printing multiple biomaterials with distinct mechanical characteristics, it is possible to recreate these gradients within a single construct, thereby more closely mimicking the hierarchical structure of native cartilage and improving functional performance after implantation.

Cartilage is composed not only of chondrocytes, but also of additional cell types that contribute to tissue development and maintenance. Fibroblasts and stem cells, for example, play vital roles in cartilage formation and repair. Accordingly, co-culture strategies incorporating multiple cell types, such as chondrocytes, fibroblasts and stem cells within bioprinted constructs may enhance tissue complexity and more effectively replicate the native cellular microenvironment. High-resolution bioprinting further enables the fabrication of intricate and spatially precise 3D architectures that resemble native cartilage. Moreover, the integration of real-time monitoring and feedback systems during printing may allow dynamic adjustments to process parameters, thereby improving construct fidelity and biological functionality.

To ensure reproducibility and biological performance, the optimization of the printing process is essential. High-precision fabrication of complex structures requires continued advancements in nozzle design, printing speed, resolution, and material deposition control (143). While current research has primarily focused on printing small tissue constructs, there is increasing interest in generating entire complex organs such as the heart and brain. Although progress has been achieved in disease modeling and partial tissue repair in organs, including complex organs such as the heart, liver, kidney, and neural tissues (135), fabrication of fully functional complex organs remains a substantial challenge. In particular, printing neural tissues such as the brain requires advanced technologies capable of monitoring functional activity.

Advancements in printing technologies are also being explored to improve structural accuracy and resolution. The incorporation of advanced imaging modalities, including CT and MRI, can facilitate the generation of detailed 3D models of affected joints, thereby enhancing the anatomical precision of printed constructs. A growing body of evidence supports the therapeutic potential of bioprinting-enabled drug delivery systems for the treatment of OA. Bioprinting allows localized and controlled drug release within the affected joint, thereby improving targeting accuracy and reducing systemic side effects. Notably, sustained drug release profiles can be achieved, maintaining therapeutic concentrations over extended periods and potentially providing prolonged symptom relief and disease-modifying effects. In addition, bioprinted constructs containing chondrocytes or MSCs have demonstrated the ability to promote cartilage-like tissue formation and support regenerative processes (145).

Preclinical studies using 3D bioprinting in rabbit models of joint regeneration have demonstrated high post-printing cell viability and functional tissue repair. For example, a GelMA scaffold containing rabbit bone marrow MSCs showed ~81% viability 48 h after bioprinting and supported cartilage regeneration in a New Zealand rabbit osteochondral defect model (146). Additionally, reviews of 3D bioprinted cartilage point out that cell viability commonly reaches ~70-85% immediately after printing and can recover to high levels approaching ~90% within days in similar hydrogel-based constructs used for orthoregeneration (147,148). Despite these encouraging results, comprehensive clinical trials are required to establish the safety, efficacy and long-term outcomes of bioprinted cartilage implants prior to routine clinical applications. The incorporation of personalized medicine strategies, including patient-specific design and fabrication, may further enhance the success rates of cartilage repair. Furthermore, long-term durability and performance of bioprinted cartilage constructs depend on the integration of materials and structural features that improve lubrication, a critical determinant of joint function.

A major challenge in bioprinting remains the development of optimal bioinks. Bioinks, composed of biomaterials, cells, or their combinations, are essential for the fabrication of structurally stable and biologically functional constructs. Despite significant technological advancements, designing bioinks that simultaneously satisfy mechanical, rheological and biological requirements remains complex. Ongoing research is therefore directed toward the development of novel bioink formulations and the establishment of standardized evaluation methods. A notable study introduced a method for assessing bioink shape fidelity by analyzing filament fusion and collapse behavior in 3D-printed structures, providing valuable insight into bioink suitability for specific applications (149).

Of note, the translation of bioprinted cartilage constructs from laboratory research to routine clinical practice will depend on overcoming several technological and regulatory barriers. Although promising results have been demonstrated *in vitro* and in preclinical animal models, large-scale clinical implementation requires rigorous evaluation of safety, long-term functionality and reproducibility. Regulatory approval processes for bioprinted tissues are particularly complex as these constructs combine living cells, biomaterials and manufacturing technologies, placing them at the intersection of medical devices, biologics and advanced therapy medicinal products. Current regulatory frameworks from agencies such as the US Food and Drug Administration (FDA) and the European Medicines Agency (EMA) require extensive preclinical validation, standardized manufacturing protocols and controlled clinical trials before approval can be granted. Considering the current pace of technological development and ongoing preclinical studies, initial human trials of MSC-laden bioprinted cartilage constructs may become feasible within the next ~5-10 years, while broader clinical adoption is expected to take longer, depending on progress in regulatory approval, manufacturing standardization, and construct maturation (147,150,151). Continued advancements in bioink development, printing resolution, construct maturation, and regulatory standardization will therefore play a crucial role in accelerating the translation of cartilage bioprinting technologies into clinical therapies (134,135,152).

## 15. Conclusion

Bioprinting provides a powerful platform for the fabrication of functional cartilage tissues through precise control of cells and biomaterials. The use of iPSCs as a patient-specific and readily available cell source further enhances the translational potential of this approach. By rationally designing bioinks with appropriate mechanical properties, incorporating specific growth factors and using cues from the native ECM, it is possible to effectively direct iPSC differentiation toward a chondrogenic lineage. To achieve long-term functional outcomes, strategies aimed at enhancing the lubrication properties of regenerated cartilage are also essential. These may include the incorporation of fluid-filled microchannels or HA within the bioprinted construct to better replicate the native joint environment. Although critical challenges remain, including the optimization of vascularization and ensuring long-term structural stability, continued research in this field holds substantial promise for the development of personalized and effective therapeutic strategies for cartilage regeneration.

## Figures and Tables

**Table I:** Comparison of cell sources used in cartilage bioprinting.

Cell type	Advantages	Disadvantages	(Refs.)
Mesenchymal stem cell (MSC)	Multipotent cells capable of chondrogenic differentiation; widely available; expandable *in vitro*	Risk of hypertrophic differentiation; donor variability	(24,35,47,48)
Induced pluripotent stem cell (iPSC)	Unlimited proliferation; patient-specific; high differentiation potential	Tumorigenic risk; complex differentiation protocols	(3,53,55,59)
Articular chondrocyte	Native cartilage-producing cells produce collagen II and proteoglycans	Limited availability; dedifferentiation during expansion	(2,4,49,50)
Articular cartilage progenitor cell (ACPC)	High proliferative capacity; strong chondrogenic potential	Limited availability; still under investigation	(18,56)

## Data Availability

Not applicable.
